# Comprehensive Review on the Protective Effects of *Lycium barbarum* Polysaccharide on Neurodegenerative Diseases

**DOI:** 10.2174/0118715303393082251027072838

**Published:** 2025-12-04

**Authors:** Qingxin Lu, Xin Di, Xiaoli Guo

**Affiliations:** 1 College of Pharmacy, Ningxia Medical University, Yinchuan 750004, China;; 2 Key Laboratory of Protection, Development and Utilization of Medicinal Resources in Liupanshan Area, Ministry of Education, Ningxia Medical University, Yinchuan 750004, China;; 3 Key Laboratory of Ningxia Minority Medicine Modernization, Ministry of Education, Ningxia Medical University, Yinchuan 750004, China

**Keywords:** Neurodegenerative disease, *Lycium barbarum* polysaccharide, alzheimer’s disease, oxidative stress, parkinson’s disease, ischemic stroke

## Abstract

Neurodegenerative diseases including Alzheimer’s disease, Parkinson’s disease, Huntington’s disease, ischemic stroke, and other related conditions, significantly impact the quality of life, particularly in low-income nations. The pathogenesis of these diseases is driven by neuroinflammation, oxidative stress, apoptosis, mitochondrial dysfunction, and regulation of autophagy. To date, no therapies or medications can fully reverse the progression of these diseases. Natural polysaccharides have demonstrated significant therapeutic potential in the treatment of neurodegenerative diseases. *Lycium barbarum* polysaccharide, derived from the traditional Chinese medicine *Lycium barbarum* L., has attracted considerable attention for its diverse biological activities, biodegradability, ease of modification, and low toxicity. This review aims to systematically evaluate the neuroprotective effects of *Lycium barbarum* polysaccharides and their mechanisms in various neurodegenerative disease models. For this study, we used multiple scientific databases, including PubMed, Scopus, Web of Science, and Google Scholar. We verified the correct plant name through the website plantlist.org. The search results were interpreted and documented based on the retrieved bibliographic information. *Lycium barbarum* polysaccharide has shown significant therapeutic potential for the treatment of neurodegenerative diseases. Experimental evidence reveals its neuroprotective effects through various mechanisms, including reducing neuroinflammation, alleviating oxidative stress, inhibiting apoptosis, improving mitochondrial function, and regulating autophagy. The current review established that *Lycium barbarum* polysaccharide holds promise as a therapeutic agent for neurodegenerative diseases. However, further research is required to address the limitations identified in previous studies and to guide future experimental investigations and clinical applications.

## 1 INTRODUCTION

Neurodegenerative disorders represent a group of debilitating illnesses, marked by the gradual and region-specific deterioration of neuronal integrity, and are associated with abnormal and heterogeneous protein accumulation [1]. Common neurodegenerative diseases can be categorized into acute and chronic. The former encompasses epilepsy, traumatic brain injury (TBI), and ischemic stroke, while the latter covers Alzheimer’s disease (AD), Parkinson’s disease (PD), Huntington’s disease (HD), and others [2]. These disorders are generally characterized by cognitive dysfunction, dyskinesia, cerebellar ataxia, limb tremor, and ocular tremor. Moreover, the prevalence rate tends to increase sharply with advancing age, burdening global public health [3-6]. Currently, therapeutic approaches for most neurodegenerative diseases are limited to symptomatic treatment and fail to control or reverse disease progression effectively. Deciphering the pathogenesis of each neurodegenerative condition remains a long-term challenge, given the limited understanding of the human brain and the heterogeneity of neurodegenerative disease pathology. Natural products are pivotal in drug development, serving as a primary source of active pharmaceutical ingredients [7]. Based on the above, natural products may be essential in treating neurodegenerative diseases. Plants, animals, and microbes all contain large amounts of natural polysaccharides, which have attracted significant interest due to their diverse biological activities and are believed to be potentially valuable for reducing neuronal damage [8].

*Lycium barbarum* L. (Solanaceae), also known as goji in China, has been a traditional medicinal food for over 2000 years. It is primarily found in East Asia, particularly in China's Ningxia Hui Autonomous Region, which is also a significant source of commercial production. Shen Nong’s Materia Medica lists the mature dried fruit of the goji berries as a prestigious herb, noted for its ability to ‘enhance tolerance to cold and heat with long-term use and delay aging’ [9]. In traditional Chinese medicine, goji berries are commonly used to alleviate the symptoms of various ailments, including blurred vision, exhaustion, infertility, dry cough, vertigo, abdominal pain, and headaches. It has been listed in the Chinese Pharmacopoeia. Goji berries comprise approximately 40% sugars by dry weight, with *Lycium barbarum* polysaccharide (LBP) making up around 5–8% [10]. The main active ingredients of goji berries include LBP, betaine, taurine, vitamins, flavonoids, alkaloids, inorganic salts, and trace minerals [10]. Among these, LBP is recognized as the most critical component due to its various pharmacological properties. As shown in Fig. (**1**), LBP comprises various monosaccharides and 17 amino acids, including xylose, glucose, and slight amounts of galactose, arabinose, mannose, rhamnose, and others [11].

**Fig. (2):**
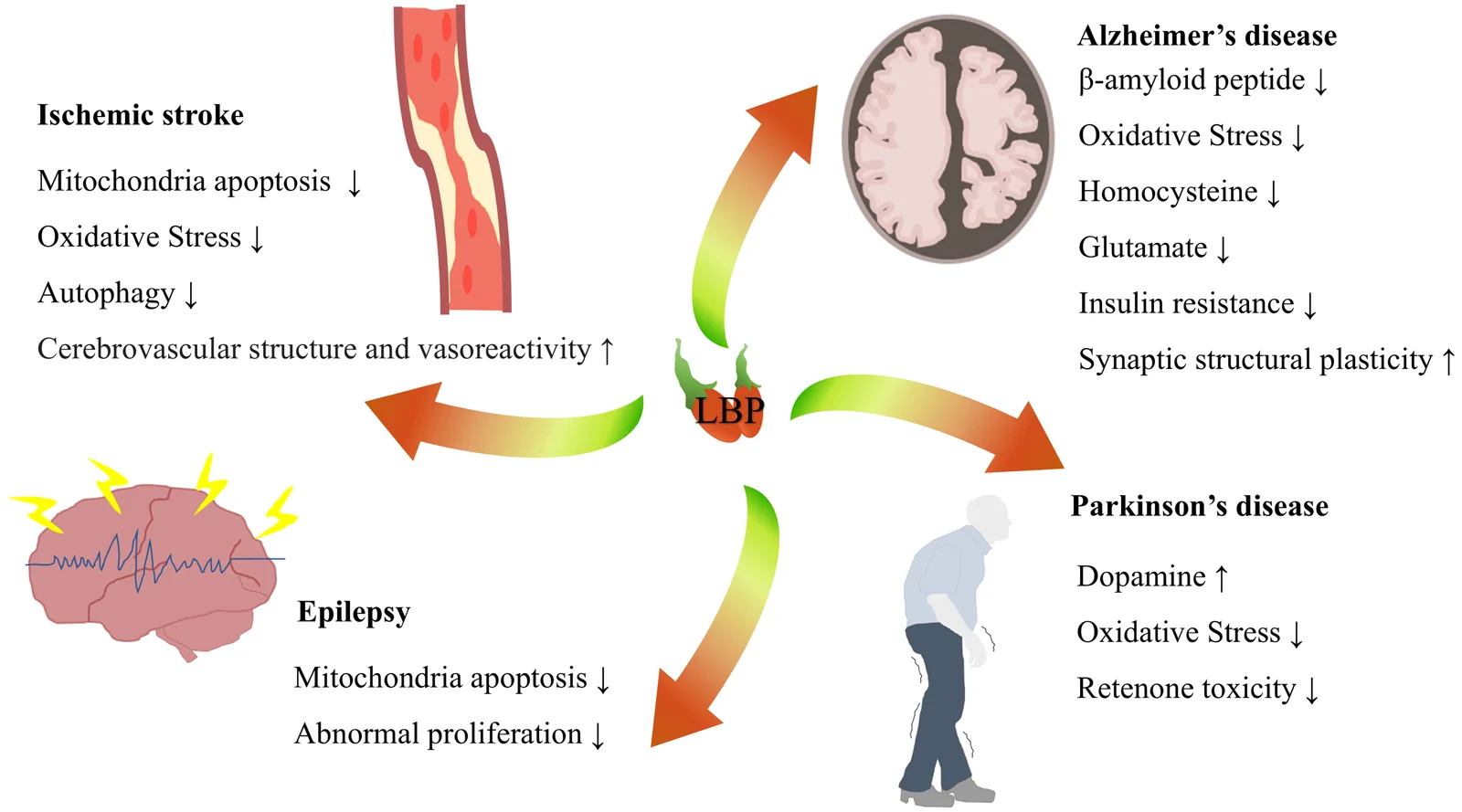
Neuroprotective properties of LBP in major neurodegenerative diseases.

LBP exhibits diverse pharmacological activities across multiple human organs, including anti-inflammatory, anti-aging, antioxidant, anti-fatigue, anticancer, antidiabetic, and immunomodulatory effects. Notably, studies have shown that LBP exerts direct neuroprotective effects in models of neurodegenerative diseases such as AD, ischemia/reperfusion injury, PD, and HD through antioxidant activity, immune regulation, inhibition of apoptosis and DNA damage [12] (Fig. **2**). Taking account of the high incidence, mortality, and consequent heavy economic burden of neurodegenerative diseases, further elucidation of precise pathologic mechanisms and the development of effective and affordable therapeutics remain significant for developing safe, viable and efficient treatments for such diseases in clinical medicine. To summarize the research on the efficacy of LBP as a supportive therapy for patients with neurodegenerative disorders and its underlying mechanisms, we conducted an integrated review.

## 2 METHODOLOGY

Scientific databases, including PubMed, Scopus, Web of Science, and Google Scholar, were searched in English until the end of December 2024. The search terms were keywords alone or in combination with each other, such as “neurodegenerative diseases” OR “Alzheimer's disease” OR “Parkinson's disease” OR “Huntington's disease” OR “ischemic stroke” OR “neuroinflammation” OR “oxidative stress” OR “apoptosis” OR “*Lycium barbarum* polysaccharide” OR “neuroprotective effects” OR “therapeutic potential” OR “biological activities” OR “biodegradability” AND “*Lycium barbarum* L.”. We verified the correct plant name through the website plantlist.org.

## 3 LBP’S MAJOR POLYSACCHARIDE CLASSES AND EXTRACTION METHODS

Established methodologies exist for the separation and structural characterization of purified LBPs, bioactive constituents exhibiting diverse biological functions. Over 30 distinct polysaccharides have been identified from *L. barbarum* L., characterized primarily through: (1) HPGPC for molecular weight/homogeneity determination; (2) monosaccharide composition analysis *via* partial acid/enzymatic hydrolysis, periodate oxidation, Smith degradation, HPLC, GC, PACE, and HPTLC; (3) 1H/13C NMR spectroscopy for assigning monosaccharide ratios and anomeric bonds; (4) IR spectroscopy for identifying pyranose/furanose rings and anomeric configurations; and (5) GC-MS for linkage position analysis [13]. Extensive studies confirm LBPs are acidic heteropolysaccharides (10-2300 kDa) containing polypeptide/protein components, purified using gel-permeation chromatography and HPLC. Approximately 20 constituent monosaccharides have been characterized, with structural analyses revealing glycoconjugate architectures defined by amino acid residues, branching patterns, side-chain configurations, and glycan backbone glycosidic linkages. Table **1** summarizes documented polysaccharide structures, molecular weights, and bioactivities in *L. barbarum*.

Extraction methods for LBPs exhibit distinct advantages and limitations [13]. Hot water extraction (HWE), the conventional technique, shows yield variability that is highly dependent on extraction duration, temperature, and water-to-material ratio. Prolonged high-temperature processing may induce polysaccharide degradation, potentially compromising bioactivity. Enzyme-assisted extraction (EAM) offers environmental sustainability, operational efficiency, minimal energy requirements, and cost-effectiveness; however, enzymatic specificity and sensitivity to concentration, temperature, pH, and processing time can impact polysaccharide functionality. Microwave-assisted extraction (MAE) provides enhanced yield, reduced solvent consumption, shorter extraction time, and operational economy. Ultrasonic-assisted extraction (UAE) improves solvent penetration *via* capillary effects, thereby increasing extraction efficiency; however, ultrasonic energy may alter polysaccharide molecular weight and structural integrity, thereby affecting biological activity. Comparative extraction parameters and yields across these methodologies are summarized in Table **2**.

## 4 LBP AND NEURODEGENERATIVE DISEASES

### 4.1 LBP and AD

AD, the most prevalent neurodegenerative disease, affects approximately 40 million people globally. Due to the aging population, the number of cases is projected to reach 139 million by 2050 [21]. The amyloid cascade hypothesis is the most widely recognized explanation of AD pathogenesis. By definition, AD is a form of dementia with the deposition of amyloid plaques [22], mainly composed of Aβ. Furthermore, this hypothesis asserts that neuronal and synaptic toxicity is attributed not to amyloid plaques but to the accumulation of toxic Aβ oligomers. Therefore, for over 30 years, targeting the reduction of Aβ production or enhancing its clearance from the patient’s brain has been a primary objective in developing novel therapeutics for AD [23].

LBPs extracted by distinct methods had different structures and molecular weights [24]. The molecular weight of LBP ranged from 10 to 2300 kDa [25]. LBPs of various molecular weights (LBP1 < 10 kDa, 10 kDa < LBP2 < 30 kDa, 30 kDa < LBP3 < 50 kDa, and LBP4 > 50 kDa) play different protective roles in neurodegenerative diseases. Zhou *et al*. found [14] that LBP1 (molecular weight 3.35 kDa) may reduce Aβ production in neuronal cells and AD cell models by affecting Aβ processing *in vitro*. The results of in vivo experiments further suggested enhanced cognitive abilities and ameliorated synaptic dysfunction in the hippocampal CA3-CA1 pathway in APP/PS1 transgenic mice. The crude polysaccharide LBP1 was isolated and purified to yield LBP1A1-1 and LBP1C-2, which may be lead compounds for treating AD. LBP1A1-1 not only directly blocked the production of Aβ42 but also dose-dependently inhibited the aggregation of Aβ42 and increased the content of Aβ42 monomer, suggesting that LBP1A1-1 may have some depolymerizing effects [26]. Similarly, LBP1C-2 can inhibit the aggregation of Aβ42 and reduce Aβ by inhibiting the metabolism of APP *via* the amyloid pathway and promoting the metabolism of APP *via* the “non-amyloid pathway” [27]. Modulating the activity of β-secretase and γ-secretase to decrease the Aβ42/Aβ40 ratio has emerged as a novel therapeutic strategy for AD [28]. Wu *et al*. demonstrated that LBP-3 treatment could exert neuroprotective effects by altering the expression levels of 402 proteins, including APH-1, caspase, and other differentially accumulated proteins (DAPs), thereby reducing Aβ42/Aβ40 levels in N2a/APP695 cells [15]. In addition, LBP pretreatment effectively protected neurons from Aβ-induced apoptosis by reducing caspase-3 and caspase-2 activity [29]. Three different fractions of LBP were obtained after semi-purification: LBP-I, LBP-II, and LBP-III. Interestingly, only LBP-III showed a neuroprotective effect against Aβ toxicity in the caspase-3 activity assay. Since activated caspase-3 affects neuronal cell death and NFT and plaque formation in AD brain [30], and caspase-2 mediates Aβ-induced neuronal cell death on Aβ neurotoxicity, LBP-III may be a potential neuroprotective agent for treating AD [31].

Aging is the most severe risk factor identified for AD. Oxidative stress, a critical component of the aging process, connects various hypotheses and mechanisms in AD. It promotes AD progression by increasing Aβ deposition, tau hyperphosphorylation, and subsequent synaptic and neuronal loss [32, 33].

*Lycium barbarum* L. remains a commonly used anti-aging drug in Chinese medicine. LBP, its primary ingredient, has been shown in numerous investigations to be a powerful antioxidant and anti-aging agent [34]. Additionally, oral administration of LBP has been shown to reduce the production of MDA, an essential end-product of oxidative stress, in aged mice’s blood and other organs [35], thereby reversing elevated endogenous lipid peroxidation and enhancing antioxidant activity. Moreover, LBP restored compromised immune parameters, including thymic and splenic indices and phagocytic activity, in aged mice [35]. By sequentially exposing zebrafish embryos to different concentrations of LBP, Xia *et al*. [36] demonstrated that sequential exposure of zebrafish embryos to varying concentrations of LBP enhanced resistance to replicative senescence, particularly at the 3.0 mg/mL concentration. Along with the diminished expression of genes associated with the regulation of senescence (*e.g*., p53, p21, Bax) [37], it can be speculated that part of the favorable impact of LBP on apoptosis and senescence may be directed through the p53-mediated signaling pathway [36]. Recent research has demonstrated that LBP activates the Nrf2/HO-1 signaling pathway to counteract oxidative stress-related neurotoxicity in various models. LBP not only inhibits the increase of reactive oxygen species (ROS) induced by H2O2 and mitochondrial apoptosis in PC12 cells [38] but also improves cognitive function in light-stimulated mice and alleviates apoptosis, oxidative stress, and mitochondrial damage in HT-22 cells [39]. Based on its antioxidant properties, LBP may attenuate oxidative stress and reduce aging-related neuronal degeneration.

Homocysteine (Hcy), a sulfur-bearing, non-proteinogenic α-amino acid, shares homology to the amino acid cysteine, and elevated plasma Hcy levels cause hyperhomocysteinemia (HHcy). An increasing number of studies suggest [40, 41] that HHcy-induced inflammation may induce AD, atherosclerosis, ischemic injury, and osteoarthritis in the elderly. Also, the clinical manifestations of AD-related hyperhomocysteinemia encompass the accumulation of Aβ and phosphorylated tau (p-tau) [42]. Ho *et al*. argued [43, 44] that LBP attenuates Hcy-induced increases in phosphorylated ERK-1 and JNK-1 while attenuating pathogenic alterations such as apoptosis, tau phosphorylation, and tau fragmentation in primary cortical neurons. The beneficial effect of LBP in homocysteine neurotoxicity further supports the idea of its use as an antidote for AD therapeutic intervention. However, LBP intake did not affect serum homocysteine levels in obese rats [45]. Thus, it is speculated that the anti-homocysteinemia effect should be specific to LBP.

Glutamate is an excitatory neurotransmitter that regulates neurotransmission, synaptogenesis, neuronal plasticity, learning, and memory by activating glutamatergic receptors [46]. In the AD brain, Aβ may prevent astrocytes from effectively clearing glutamate, leading to neuronal enlargement, breakdown of cell membrane integrity, and ultimately cell death [47]. Therefore, dysfunction of the glutamatergic system is considered one of the core characteristics of AD, and the existing AD prescription drug, memantine, is developed to target the glutamatergic system [46].

LBP inhibits glutamate-induced cell death and induces JNK phosphorylation and attenuates n-methyl-d-aspartate (NMDA)-induced neuronal damage [48]. LBPS02, purified from LBP, protects against apoptosis in differentiated PC12 cells because of its ability to repair impaired L-Glu homeostasis. Meanwhile, it has been shown that LBPS02-mediated neuroprotection is also closely associated with regulating extracellular signal-regulated kinases (ERKs), phosphorylation of protein kinase B (AKT), and secondary improvement of mitochondrial dysfunction [16].

Impaired insulin response in brain cells, leading to glucose intolerance and hyperglycemia, contributes to the development and advancement of AD [49]. In a clinical trial by Cai *et al*., LBP reduced blood glucose levels and increased the insulinogenic index in individuals diagnosed with type 2 diabetes [50]. Within a streptozotocin (STZ)-induced diabetic rat model, LBP decreased serum amyloid A3 expression by inhibiting NF-κB activation. Furthermore, in an STZ-induced AD model, LBP exerted neuroprotective effects by regulating the IRS1/PI3K/AKT pathway, suppressing aberrant tau phosphorylation, and upregulating synapse-related protein expression [51].

### 4.2 LBP and Ischemic Stroke

Neurodegenerative diseases are broadly categorized into acute and chronic subtypes, depending on the temporal progression and underlying pathophysiology [52, 53]. Emerging literature supports the inclusion of stroke within the framework of acute neurodegenerative disorders [2, 54]. As reported in the World Stroke Organization’s Global Stroke Fact Sheet, stroke endures as the second-leading cause of death and the third-leading cause of death and disability combined [55]. Unfortunately, the stroke burden is predicted to continue to rise worldwide, particularly in low-income countries [56]. Although many stroke patients survive, they often suffer from long-term disabilities. A stroke leads to brain cell death due to impaired oxygen and nutrient supply and degradation in crucial brain functions, including memory and muscle control [57]. Ischemic strokes account for the majority of strokes. The known processes underlying stroke include vascular embolism, neuro-oxidative stress, inflammation and apoptosis, decreased synaptic transmission, excitotoxicity, and aberrant expression/activity of numerous channels and signaling proteins [58]. It is now recognized that timely restoration of blood flow to ischemic brain tissue is essential. A broad range of natural polysaccharides has garnered significant interest in stroke drug development because of the ability to inhibit permanent damage to brain tissue caused by intracellular ROS and RNS production, intracellular Ca2+ overload, glutamate neurotoxicity, inflammation, and apoptosis, pathological responses that occur during ischemia or reperfusion [59, 60].

Professor Yu’s research group investigated various aspects of LBP’s anti-ischemic stroke mechanism. They initially reported [61] that LBP dose-dependently prevented oxygen-glucose deprivation/reperfusion (OGD/R) damage in primary cultured rat hippocampal neurons through anti-oxidative stress, maintenance of mitochondrial membrane potential (MMP), and inhibition of intracellular Ca2+ increase. Consistently, the combination of ω-3 PUFAs and LBP has been shown further to provide enhanced protection for cortical neurons against OGD/R injury [62].

Increasing evidence suggests various cell death mechanisms, including apoptosis, autophagy, pyroptosis, and ferroptosis, are associated with cerebral ischemia-reperfusion injury [63]. Given apoptosis’s role in stroke, the effects of LBP have been extensively studied. Yang *et al*. demonstrated [64] that oral LBP administration reduced brain apoptosis, improved hemispheric swelling and water content in mice with transient middle cerebral artery occlusion (MCAO), and significantly reduced blood-brain barrier (BBB) disruption induced by aquaporin-4 (AQP-4) and glial cell activation induced by glial fibrillary acidic protein. Subsequently, Wang *et al*. found [65] that by preventing the overexpression of Bax, CytC, Caspase-3, Caspase-9, and PARP-1, LBP also inhibited the mitochondrial apoptotic pathway, which in turn defended against focal cerebral ischemia injury. In addition, LBP has been shown to inhibit N-methyl-D-aspartate receptor 2B(NR2B)-mediated autophagy, which can alleviate glutamate-mediated excitotoxicity [66]. Autophagy plays a dual role in ischemic stroke, acting as a protective mechanism for cell survival by reducing neuronal loss yet potentially leading to cell death and exacerbating brain injury when excessively activated [67]. Yu *et al*. argued [68] that LBP could upregulate Bcl-2/Bax and p62 and downregulate cleaved Caspase-3/Caspase-3, LC3II/LC3I, and Beclin 1. That is, LBP protects primary hippocampal neurons from OGD/R injury by activating the PI3K/AKT/mTOR signaling pathway, thereby inhibiting apoptosis and autophagic cell death [68]. In addition, recent studies have shown that LBP inhibits ischemia-induced autophagy by enhancing miR-133a-3p delivery by promoting the biogenesis of extracellular vesicles derived from neural stem cells [69].

Modulating neuroinflammation is profoundly significant for improving long-term outcomes and alleviating comorbidities in stroke patients [70]. Another research by Zhao *et al*. illustrated that [71] LBP enhanced spatial memory and motor coordination in mice suffering from ischemic stroke. This improvement was partially attributed to LBP’s capacity not only to inhibit MCAO-induced activation of P65 NF-κB and P38 MAPK but also to reverse the upregulation of proinflammatory mediators in the hippocampus. Additionally, LBP influenced the TLR4/NF-κB signaling pathway, which had antioxidant and anti-inflammatory effects while increasing brain cell viability and reducing morphological cell damage [72].

According to research by Zhang’s research team, LBP exerts a significant ameliorative effect on ischemic injury exacerbated by hyperglycemia. At first, LBP reversed the hyperglycemia-induced increase in phosphorylated Drp1 and decreased Opa1, maintained dynamic mitochondrial homeostasis, and improved ischemic brain injury caused by enhanced hyperglycemia [73]. Secondly, LBP treatment preserved cerebrovascular integrity and vasoreactivity by reducing vascular wall thickness and increasing the expression of α-smooth muscle actin (α-SMA) and endothelial nitric oxide synthase (eNOS), as well as the cross-sectional area (CSA) of cerebral vessels [74]. Lastly, LBP mitigates hyperglycemia-induced blood-brain barrier disruptions, including endothelial cell swelling, decreased expression of tight junction proteins, and elevated IgG permeability [75].

### 4.3 LBP and Epilepsy

Epilepsy is a widespread, severe, and persistent neurological disorder affecting the lives and health of over 70 million people worldwide [76]. While epilepsy is traditionally classified as a neurological disorder characterized by transient neuronal hyperexcitability, emerging evidence highlights its chronic neurodegenerative aspects, particularly in the context of prolonged or recurrent seizures [77]. Epilepsy is commonly associated with some neurodegenerative and pathological alterations in those areas of the brain that are involved in repeated electrographic seizures. In animal models in which seizures are either repeatedly elicited or are self-generated, a similar set of neurodegenerative and pathological alterations in brain anatomy is observed. Therefore, based on the contemporary definition established in recent literature [78, 79], epilepsy is incorporated into the discourse on neurodegenerative diseases.

Currently, Central Nervous System (CNS) infections, genetic disorders, brain masses, and brain injuries are recognized as the primary etiologies of epilepsy. Although the pathogenesis of epilepsy has been dissected at the genetic level, both the frequent occurrence of drug resistance events and the fact that approximately 30-40% of patients experience adverse reactions to existing antiepileptic drugs have made the identification of new antiepileptic components from natural medicines a new strategy today [80, 81]. Zhang *et al*. found [82] that LBP possessed a neuroprotective effect by reducing the abnormal proliferation of BrdU-positive cells in the granular layer of the hippocampal dentate gyrus after seizures in rats. The experimental results of Ma *et al*. showed [83] that LBP exerted on an antiepileptic effect by down-regulating the mitochondrial apoptotic pathway. Pro-inflammatory cytokines, such as IL-1β and TNF-α, play a pivotal role in driving neuroinflammation and neuronal hyperexcitability in epilepsy. These mediators are not only consequences of seizures but also actively contribute to epileptogenesis (the development of epilepsy) and the perpetuation of chronic seizure activity. They disrupt neuronal networks and glial homeostasis. In epileptic cells, LBP additionally suppresses the expression of inflammatory mediators, including IL-1, IL-1β, and TNF-α [83].

### 4.4 LBP and PD

PD manifests as motor bradykinesia accompanied by resting tremor, rigidity, or both [84], resulting from multifaceted interactions among environmental and genetic factors, including neuroinflammation, protein aggregation, oxidative stress, impaired autophagy, and mitochondrial impairment [85]. LBP has demonstrated neuroprotective effects in PD models, such as 1-Methyl-4-phenyl-1,2,3,6-tetrahydropyridine(MPTP)-induced rodent and silkworm models. LBP improves motor function, restores dopamine levels, and enhances tyrosine hydroxylase activity, which is crucial for dopamine synthesis [86]. It also alleviates oxidative stress by upregulating antioxidant enzymes (SOD2, CAT, GPX1) and reduces α-synuclein aggregation [87]. Furthermore, LBP activates the PTEN/AKT/mTOR signaling pathway, indicating its potential to protect dopaminergic neurons [87]. Through the ROS-NO pathway, LBP also prevents 6-OHDA-induced apoptosis [88]. These findings position LBP as a potential therapeutic agent for PD, targeting both neurodegeneration and oxidative damage.

### 4.5 LBP and HD

The main features of HD, also known as Huntington’s chorea, include pronounced involuntary motor abnormalities, psychiatric symptoms, and cognitive decline, which result from the loss of neurons in the striatum of patients [89]. The trinucleotide repeat expansion in exon 1 of the HD gene, which encodes HTT, is the underlying cause of this progressive neurological disease [89, 90]. Consequently, viable therapeutic approaches to reduce the expression of HTT protein mutants (mHTT) have been sought for many years [91, 92]. Studies have shown that LBP alleviates mHTT-induced cytotoxicity by activating AKT signaling and decreasing mHTT levels in the cortex, hippocampus, and striatum of HD transgenic mice [93]. As a result, improvements in motor function and lifespan were observed in HD transgenic mice, suggesting that LBP may have therapeutic potential for HD.

To date, various studies have provided a comprehensive overview of LBP's neuroprotective mechanisms and efficacy across different neurodegenerative conditions, along with the specific disease models used. Details are provided in Fig. (**3** and Table **3**).

## CONCLUSION AND PERSPECTIVES

In summary, LBP is a potent neuroprotective agent obtained from *Lycium barbarum* L. Numerous *in vivo* and *in vitro* investigations have shown that LBP exhibits neuroprotective properties in both primary and secondary neurodegenerative disorders, including AD, ischemic stroke, epilepsy, PD, and HD [94, 95]. While the protective mechanisms of LBP vary across different conditions, it generally exerts neuroprotective effects by attenuating neuroinflammation, oxidative stress, apoptosis, and mitochondrial dysfunction, and by regulating autophagy in the brain. Notably, other bioactive polysaccharides exhibit complementary neuroprotective properties. Polysaccharides from Ecklonia maxima, Gelidium pristoides, and Ulva rigida, containing sulfate groups, inhibit cholinesterase, thereby exerting anti-AD effects [8]. Moreover, a polysaccharide derived from green algae has been shown to exhibit anti-PD activity by inhibiting α-Synuclein fibrillation [8].

More in-depth investigations into the mechanisms underlying neurodegenerative diseases have established that diet is one of the key modifiable risk factors [96]. For centuries, *Lycium barbarum* L. has served as a traditional Chinese medicine and tonic [97], and food-derived polysaccharide LBP may have potential value as a functional food supplement for neuroprotective effects in the daily diet [98]. According to the Chinese Pharmacopoeia, 86 Chinese patent medicines containing goji are currently on the market, such as Shenqi Jiangtang Tablets and Qiming Granules. The clinical efficacy of these formulations is primarily manifested as tonifying qi and nourishing yin, replenishing the kidney and augmenting essence, nourishing the liver and kidney, supplementing qi and blood, and improving vision. However, to date, no clinical studies on LBP have been reported. This gap may be attributed to the challenges posed by its diverse monosaccharide composition, chain conformation, ring configuration, and molecular weight distribution.

Moreover, distinct extraction techniques have varying implications on the quality of LBP [13] and the absence of uniform regulation of LBP sources and quality, the grade of LBP will influence the results of different research teams used [99]. Besides, the efficacy of LBP is closely correlated with its molecular weight, structure, and composition [20]. Regrettably, several published investigations did not perform quality-control tests or provide details on the purity, molecular weight, and monosaccharide composition of the LBP. Little is known about LBP’s absorption in the human body, its post-absorption distribution in organs and cells, and the products produced by the related metabolism. Furthermore, the bioavailability, optimal dosage, and therapeutic window for LBP remain unclear. Greater emphasis should be placed on clinical research investigating the therapeutic efficacy of LBP in the treatment of neurodegenerative diseases.

In the future, optimizing the drug delivery system to modulate the pharmacokinetics, efficacy, toxicity, release rate, and stability of LBP will be essential to improve its oral bioavailability and develop an oral delivery system [100, 101]. The bioavailability of LBP in different routes of administration and its safety characteristics need to be further investigated to serve clinical applications better [99]. Current studies on LBP’s role in neurodegenerative diseases remain early. The role of LBP in treating neurodegenerative disease remains to be further explored.

## Figures and Tables

**Fig. (1):**
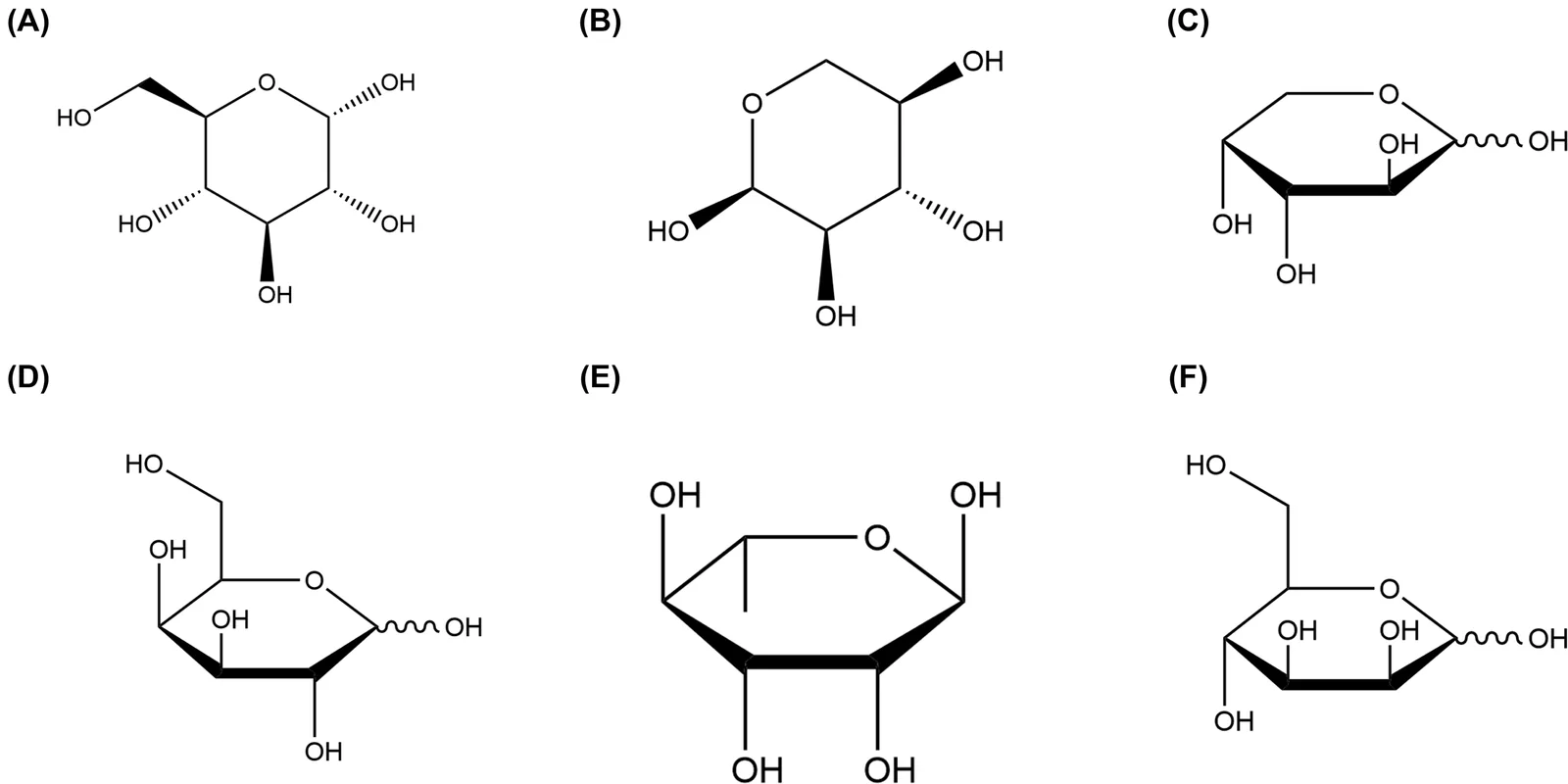
The structure of the main monosaccharides of LBP. (**A**) Glucose; (**B**) Xylose; (**C**) Arabinose; (**D**) Galactose; (**E**) Rhamnose; (**F**) Mannose.

**Fig. (3):**
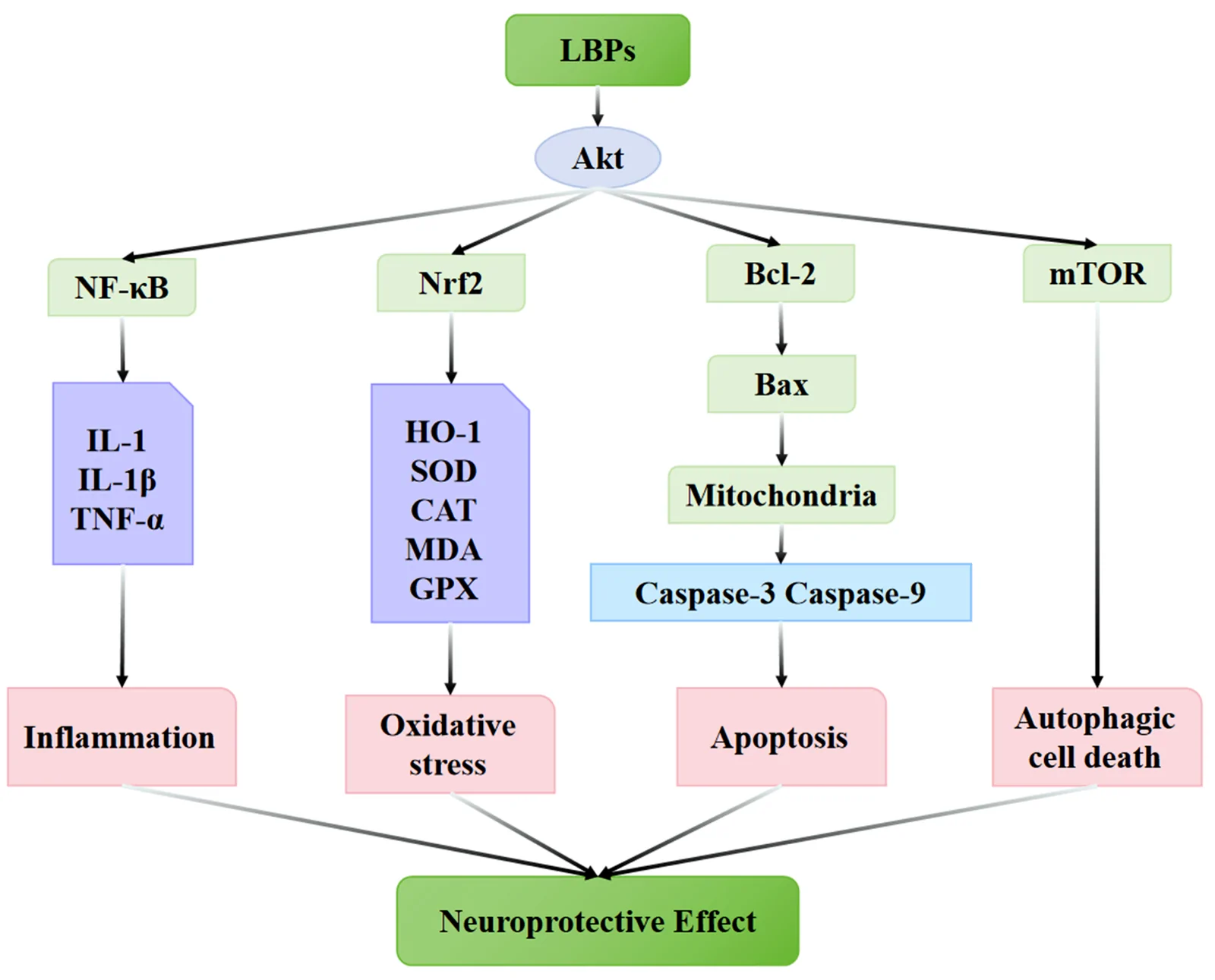
The molecular mechanisms underlying the neuroprotective activity of LBPs.

**Table1:** Structural characteristics and neuroprotective properties of common LBPs.

**LBPs**	**Mw** **(kDa)**	**Monosaccharide Composition and the Quantity Ratio of the Substance**	**Structures(Backbone)**	**Neuroprotective Effects**	**Reference**
LBP1A	3.35	Man: Glc: Gal: Xyl: Ara 6.52: 78.12:8.85:1.81: 4.69	NA	Aβ↓	[14]
LBP-3	67.4	Ara: Gal 1: 1.56	1, 3-linked β-Galp, which is partially substituted at C-6	Aβ42/Aβ40↓	[15]
LBPS02	68	NA	1 → 3, 1 → 4 and 1 → 6 linkages	Bcl-2↑, Bax↓, Cleaved Caspase-3↓	[16]
LBGP70	95	Ara: Gal: Rha: Glc 63.7: 26.8: 4.1: 2.4	(1→6)-β-Gal	SOD↑, CAT↑, GSH-Px↑, Sirt1↑, NAMPT↑, Prx1	[17]
LFP-a1	47.4	Rha: Ara: Xyl: Man: Glc: Gal: GalA 8.87: 37.17: 0.61: 0.52: 6.84: 37.20: 8.79	NA	ROS↓, Bcl-2↑, Bax↓, Caspase-3↓, SOD↑, CAT↑, GSH-Px↑,	[18]
LFP-1	17.8	Rha: Ara: Xyl: Man: Glc: Gal: GlcA: GalA 3.68: 34.88: 2.46: 1.03: 6.89: 37.64: 0.73	Significant moieties of arabinogalactan, linear homogalacturonan, and rhamnogalacturonan.	MPP+-induced neurotoxicity↓	[19]

**Abbreviations:** Ara: arabinose; Gal: galactose; GalA: galacturonic acid; Glc: glucose; GlcA: glucuronic acid; Man: mannose; NA: not mentioned; Rha: rhamnose; Xyl: xylose.

**Table 2:** Summary of the extraction methods for the extraction of LBPs.

**Extraction Methods**	**Extraction Conditions**	**Yield (%)**	**Features**	**Reference**
Water extraction method	The ratio liquid to solid is 70:1, pH 10, at 65°C, extracted in soakage for 3.5 h.	7.46–7.63%	Unstable yield	[13, 20]
Ultrasound-assisted extraction method	Extraction time of 30 min, temperature of 60°C, solid/liquid ratio of 20 g/600 mL, power density of 300 W/L, ultrasound frequency of 28 kHz.	2.286–5.701%	Higher yield and the influence on the structure and molecular weight of polysaccharides	
Enzyme-assisted extraction method	Extraction time of 91 min, extraction temperature of 59.7°C, pH 5.0.	6.81±0.10%	Higher extraction efficiency, lower investment, environmental friendliness and the influence of specificity and selectivity of enzymes	
Microwave-assisted extraction method	Ratio of water to raw material of 31.5 mL/g, extraction time of 25.8 min, microwave power of 544.0 W.	8.25±0.07%	Higher extractability and the influence on the structure and molecular weight of polysaccharides	

**Table 3:** The representative neuroprotective effects of LBP on neurodegenerative diseases.

**Disease**	**Model**	**Drug Treatment**	**Effects**	**Reference**
Alzheimer’s disease	APP/PS1 mice	Oral administration of 50 mg/kg (b.w.) or 20 g mouse body weight vehicle once a day	Enhanced neurogenesis and restored synaptic dysfunction at the hippocampus CA3-CA1 pathway	[14]
	CHO/APPBACE1 cells and HEK293-APPsw cells	Incubated with LBP1A1-1 (from 1.4 μM to 22.2 μM) for 24 h	Inhibited Aβ42 production and impeded Aβ42 aggregation	[26]
	CHO/APPBACE1 cells and HEK293-APPsw cells	Incubated with LBP1C-2 (from 0.6 μM to 10.0 μM)for 24 h	Decreased Aβ42 production	[27]
	N2a/APP695 cells	Incubated with LBP-3 (0, 1.25, 2.5, and 5 μM) for 24 h	Decreased the levels of Aβ42/Aβ40	[15]
	Aβ25-35-induced cultured neurons	Pre-treated with LBP (10, 100, and 500 μg/mL) for 1 h	Reduced the phosphorylation of PKR triggered by Aβ peptide	[29]
	Aged Kunming mice	Oral administration of LBP (200, 350, and 500 mg/kg b.w. in physiological saline)	Compensated for the decline in total antioxidant capacity, immune function, and the activities of antioxidant enzymes, and reduced the risks of lipid peroxidation	[34]
	Zebrafish embryos	Continuously exposed to LBPs (1.0, 2.0, 3.0, and 4.0 mg/mL) for 3 days	Reduced the expression level of p53, p21, and Bax genes and increased the expression of Mdm2 and TERT genes	[36]
	Cortical neurons exposed to glutamate and NMDA	Pre-treated with LBP (10 to 500 µg/mL) for 1 h	Reduced glutamate-induced cell death, attenuated NMDA-induced neuronal damage, and reduced phosphorylation of JNK with LBP	[48]
Ischemic stroke	Primary cultured rat hippocampal neurons exposed to OGD/R	Pre-treated with LBP (final concentration of 10, 20, 40 mg/L)	Anti-oxidative stress, stabilized MMP and suppressed the intracellular Ca2+ elevation	[61]
	MCAO C57BL/6N male mice	Fed with LBP (1 or 10 mg/kg) daily for 7 days	Decreased infarct size and cerebral edema and reversed the d disruption, AQP-4 up-regulation, and glial activation	[64]
	MCAO Male ICR mice	Pre-treated with LBP (10, 20 and 40 mg/kg)	Attenuating the mitochondrial apoptosis pathway	[65]
	MCAO mice	7 successive days of pretreatment with LBP (10, 20, and 40 mg/kg)	Improved spatial memory and motor coordination function by inhibited expression and activation of P65 NF-κB and P38 MAPK	[71]
Epilepsy	Lithium-pilocarpine-induced Male Sprague-Dawley rats	Administered with LBP (25 mg/kg, 50 mg/kg, 100 mg/kg) *via* oral gavage once a day for two weeks	Decrease the abnormal proliferation of BrdU-positive cells of the hippocampal dentate gyrus granular cell layer	[82]
	Rat hippocampal neurons	Pre-treated with LBP (100, 50 and 25 mg·L-1)	Downregulating the mitochondrial apoptosis pathway and the expression of inflammatory factors	[83]
Parkinson’s disease	MPTP mouse model	Injected 100 or 200 mg/kg LBP solution intraperitoneally per day	Improved motor function, upregulated antioxidant enzymes, inhibited abnormal aggregation of α-synuclein and activated PTEN/AKT/mTOR signaling pathway	[87]
	MPTP silkworm model	Fed with LBP (50, 100, and 200 mg/kg)	Improved motor ability, dopamine levels and tyrosine hydroxylase activity, and reduced oxidative damage	[86]
Huntington’s disease	B6C3-Tg(HD82Gln)81Dbo/J) mice and HEK293 cells	Intragastrically administered with LBP (40 mg/kg body weight per day at a volume of 0.1-0.2 mL/g) and cells treated with 9.6 μg/μL LBP	Alleviates the cytotoxicity of mHTT by activating AKT and reducing mHTT levels	[93]

## LIST OF ABBREVIATIONS

AD: Alzheimer’s Disease
PD: Parkinson’s Disease
HD: Huntington’s Disease
LBP: *Lycium Barbarum* Polysaccharide
APP: Amyloid Precursor Protein
Aβ: β-Amyloid
BACE1: Beta-Site APP Cleaving Enzyme 1
CHO: Chinese Hamster Ovary
PKR: Protein Kinase R
ERK: Extracellular Signal-Regulated Kinase
JNK: c-Jun N-Terminal Kinase
Nrf2: Nuclear Factor Erythroid 2-Related Factor 2
HO-1: Heme Oxygenase-1
Hcy: Homocysteine
HHcy: Hyperhomocysteinemia
NMDA: N-Methyl-D-Aspartate
STZ: Streptozotocin
IRS1: Insulin Receptor Substrate 1
PI3K: Phosphoinositide 3-Kinase
AKT: Protein Kinase B
mTOR: Mammalian Target of Rapamycin
AMPK: AMP-Activated Protein Kinase
MCAO: Middle Cerebral Artery Occlusion
OGD/R: Oxygen-Glucose Deprivation/Reperfusion
MMP: Mitochondrial Membrane Potential
AQP-4: Aquaporin-4
CytC: Cytochrome C
PARP-1: Poly (ADP-Ribose) Polymerase 1
LC3: Microtubule-Associated Protein 1A/1B-Light Chain 3
NF-κB: Nuclear Factor Kappa B
α-SMA: α-Smooth Muscle Actin
eNOS: Endothelial Nitric Oxide Synthase
BrdU: Bromodeoxyuridine
IL-1β: Interleukin-1 Beta
TNF-α: Tumor Necrosis Factor Alpha
MPTP: 1-Methyl-4-Phenyl-1,2,3,6-Tetrahydropyridine
SOD2: Superoxide Dismutase 2
CAT: Catalase
GPX1: Glutathione Peroxidase 1
PTEN: Phosphatase and Tensin Homolog
6-OHDA: 6-Hydroxydopamine
HTT: Huntingtin
mHTT: Mutant HTT
TLR4: Toll-Like Receptor 4

## References

[1] Wilson D.M., Cookson M.R., Van Den Bosch L., Zetterberg H., Holtzman D.M., Dewachter I. (2023). Hallmarks of neurodegenerative diseases.. Cell.

[2] Hussain G., Rasul A., Anwar H., Aziz N., Razzaq A., Wei W., Ali M., Li J., Li X. (2018). Role of plant derived alkaloids and their mechanism in neurodegenerative disorders.. Int. J. Biol. Sci..

[3] Gaitatzis A., Majeed A. (2023). Multimorbidity in people with epilepsy.. Seizure.

[4] Rodríguez-Santana I., Mestre T., Squitieri F., Willock R., Arnesen A., Clarke A., D’Alessio B., Fisher A., Fuller R., Hamilton J.L., Hubberstey H., Stanley C., Vetter L., Winkelmann M., Doherty M., Wu Y., Finnegan A., Frank S. (2023). Economic burden of Huntington disease in Europe and the USA: Results from the Huntington’s Disease Burden of Illness study.. Eur. J. Neurol..

[5] Aamodt W.W., Kluger B.M., Mirham M., Job A., Lettenberger S.E., Mosley P.E., Seshadri S. (2024). Caregiver burden in parkinson disease: A scoping review of the literature from 2017-2022.. J. Geriatr. Psychiatry Neurol..

[6] Ren R., Qi J., Lin S., Liu X., Yin P., Wang Z., Tang R., Wang J., Huang Q., Li J., Xie X., Hu Y., Cui S., Zhu Y., Yu X., Wang P., Zhu Y., Wang Y., Huang Y., Hu Y., Wang Y., Li C., Zhou M., Wang G. (2022). The china alzheimer report 2022.. Gen. Psychiatr..

[7] Cui D., Chen Y., Ye B., Guo W., Wang D., He J. (2023). Natural products for the treatment of neurodegenerative diseases.. Phytomedicine.

[8] Dhahri M., Alghrably M., Mohammed H.A., Badshah S.L., Noreen N., Mouffouk F., Rayyan S., Qureshi K.A., Mahmood D., Lachowicz J.I., Jaremko M., Emwas A.H. (2021). Natural polysaccharides as preventive and therapeutic horizon for neurodegenerative diseases.. Pharmaceutics.

[9] Xiao Z., Deng Q., Zhou W., Zhang Y. (2022). Immune activities of polysaccharides isolated from *Lycium barbarum* L. What do we know so far?. Pharmacol. Ther..

[10] Qiang X., Xia T., Geng B., Zhao M., Li X., Zheng Y., Wang M. (2023). Bioactive components of *Lycium barbarum* and deep-processing fermentation products.. Molecules.

[11] Teixeira F., Silva A.M., Delerue-Matos C., Rodrigues F. (2023). *Lycium barbarum* berries (Solanaceae) as source of bioactive compounds for healthy purposes: A review.. Int. J. Mol. Sci..

[12] Gao Y., Wei Y., Wang Y., Gao F., Chen Z. (2017). *Lycium Barbarum*: A traditional chinese herb and a promising anti-aging agent.. Aging Dis..

[13] Tian X., Liang T., Liu Y., Ding G., Zhang F., Ma Z. (2019). Extraction, structural characterization, and biological functions of *Lycium Barbarum* polysaccharides: A review.. Biomolecules.

[14] Zhou Y., Duan Y., Huang S., Zhou X., Zhou L., Hu T., Yang Y., Lu J., Ding K., Guo D., Cao X., Pei G. (2020). Polysaccharides from *Lycium barbarum* ameliorate amyloid pathology and cognitive functions in APP/PS1 transgenic mice.. Int. J. Biol. Macromol..

[15] Wu J., Chen T., Wan F., Wang J., Li X., Li W., Ma L. (2021). Structural characterization of a polysaccharide from *Lycium barbarum* and its neuroprotective effect against β-amyloid peptide neurotoxicity.. Int. J. Biol. Macromol..

[16] Kou L., Du M., Zhang C., Dai Z., Li X., Zhang B., Hu X. (2017). Polysaccharide purified from *Lycium barbarum* protects differentiated PC12 cells against L-Glu-induced toxicity *via* the mitochondria-associated pathway.. Mol. Med. Rep..

[17] Huang W., Zhao M., Wang X., Tian Y., Wang C., Sun J., Wang Z., Gong G., Huang L. (2022). Revisiting the structure of arabinogalactan from *Lycium barbarum* and the impact of its side chain on anti-ageing activity.. Carbohydr. Polym..

[18] Zhang F., Zhang X., Gu Y., Wang M., Guo S., Liu J., Xiaofei Z., Zhao Z., Qian B., Yan Y., Yu L., Xu C., Liu C., Cao F., Qian D., Duan J. (2021). Hepatoprotection of *lycii fructus* polysaccharide against oxidative stress in hepatocytes and larval zebrafish.. Oxid. Med. Cell. Longev..

[19] Zhang F., Zhang X., Guo S., Cao F., Xiaofei Z., Wang Y., Liu J., Qian B., Yan Y., Chen P., Xu C., Liu C., Qian D., Duan J. (2020). An acidic heteropolysaccharide from *Lycii fructus*: Purification, characterization, neurotrophic and neuroprotective activities in vitro.. Carbohydr. Polym..

[20] Wang J., Li S., Zhang H., Zhang X. (2024). A review of *Lycium barbarum* polysaccharides: Extraction, purification, structural-property relationships, and bioactive molecular mechanisms.. Carbohydr. Res..

[21] Reitz C., Pericak-Vance M.A., Foroud T., Mayeux R. (2023). A global view of the genetic basis of Alzheimer disease.. Nat. Rev. Neurol..

[22] Gaugler J., James B., Johnson T. (2022). 2022 Alzheimer’s disease facts and figures.. Alzheimers Dement..

[23] Bezprozvanny I. (2022). Alzheimer’s disease – Where do we go from here?. Biochem. Biophys. Res. Commun..

[24] Deng X., Li X., Luo S., Zheng Y., Luo X., Zhou L. (2017). Antitumor activity of *Lycium barbarum* polysaccharides with different molecular weights: An *in vitro* and *in vivo* study.. Food Nutr. Res..

[25] Jin M., Huang Q., Zhao K., Shang P. (2013). Biological activities and potential health benefit effects of polysaccharides isolated from *Lycium barbarum* L.. Int. J. Biol. Macromol..

[26] Zhou L., Liao W., Chen X., Yue H., Li S., Ding K. (2018). An arabinogalactan from fruits of *Lycium barbarum* L. inhibits production and aggregation of Aβ_42_.. Carbohydr. Polym..

[27] Zhou L., Liao W., Zeng H., Yao Y., Chen X., Ding K. (2018). A pectin from fruits of *Lycium barbarum* L. decreases β-amyloid peptide production through modulating APP processing.. Carbohydr. Polym..

[28] Siegel G., Gerber H., Koch P., Bruestle O., Fraering P.C., Rajendran L. (2017). The Alzheimer’s disease γ-secretase generates higher 42:40 ratios for β-amyloid than for p3 peptides.. Cell Rep..

[29] Yu M.S., Lai C.S., Ho Y.S., Zee S.Y., So K.F., Yuen W.H., Chang R.C. (2007). Characterization of the effects of anti-aging medicine Fructus lycii on beta-amyloid peptide neurotoxicity.. Int. J. Mol. Med..

[30] Su J.H., Zhao M., Anderson A.J., Srinivasan A., Cotman C.W. (2001). Activated caspase-3 expression in Alzheimer’s and aged control brain: Correlation with Alzheimer pathology.. Brain Res..

[31] Troy C.M., Rabacchi S.A., Friedman W.J., Frappier T.F., Brown K., Shelanski M.L. (2000). Caspase-2 mediates neuronal cell death induced by beta-amyloid.. J. Neurosci..

[32] Chen Z., Zhong C. (2014). Oxidative stress in Alzheimer’s disease.. Neurosci. Bull..

[33] Bai R., Guo J., Ye X.Y., Xie Y., Xie T. (2022). Oxidative stress: The core pathogenesis and mechanism of Alzheimer’s disease.. Ageing Res. Rev..

[34] Chang R.C.C., So K.F. (2008). Use of anti-aging herbal medicine, *Lycium barbarum*, against aging-associated diseases. What do we know so far?. Cell. Mol. Neurobiol..

[35] Li X.M., Ma Y.L., Liu X.J. (2007). Effect of the *Lycium barbarum* polysaccharides on age-related oxidative stress in aged mice.. J. Ethnopharmacol..

[36] Xia G., Xin N., Liu W., Yao H., Hou Y., Qi J. (2014). Inhibitory effect of *Lycium barbarum* polysaccharides on cell apoptosis and senescence is potentially mediated by the p53 signaling pathway.. Mol. Med. Rep..

[37] Rufini A., Tucci P., Celardo I., Melino G. (2013). Senescence and aging: The critical roles of p53.. Oncogene.

[38] Cao S., Du J., Hei Q. (2017). *Lycium barbarum* polysaccharide protects against neurotoxicity *via* the Nrf2-HO-1 pathway.. Exp. Ther. Med..

[39] Yang Y., Yu L., Zhu T., Xu S., He J., Mao N., Liu Z., Wang D. (2023). Neuroprotective effects of *Lycium barbarum* polysaccharide on light-induced oxidative stress and mitochondrial damage *via* the Nrf2/HO-1 pathway in mouse hippocampal neurons.. Int. J. Biol. Macromol..

[40] Farkas M., Keskitalo S., Smith D.E.C., Bain N., Semmler A., Ineichen B., Smulders Y., Blom H., Kulic L., Linnebank M. (2013). Hyperhomocysteinemia in Alzheimer’s disease: The hen and the egg?. J. Alzheimers Dis..

[41] Tawfik A., Elsherbiny N.M., Zaidi Y., Rajpurohit P. (2021). Homocysteine and age-related central nervous system diseases: Role of inflammation.. Int. J. Mol. Sci..

[42] Popp J., Lewczuk P., Linnebank M., Cvetanovska G., Smulders Y., Kölsch H., Frommann I., Kornhuber J., Maier W., Jessen F. (2009). Homocysteine metabolism and cerebrospinal fluid markers for Alzheimer’s disease.. J. Alzheimers Dis..

[43] Ho Y.S., Yu M.S., Yang X.F., So K.F., Yuen W.H., Chang R.C.C. (2010). Neuroprotective effects of polysaccharides from wolfberry, the fruits of *Lycium barbarum*, against homocysteine-induced toxicity in rat cortical neurons.. J. Alzheimers Dis..

[44] Ho Y.S., Yang X., Lau J.C.F., Hung C.H.L., Wuwongse S., Zhang Q., Wang J., Baum L., So K.F., Chang R.C.C. (2012). Endoplasmic reticulum stress induces tau pathology and forms a vicious cycle: Implication in Alzheimer’s disease pathogenesis.. J. Alzheimers Dis..

[45] Kang M.H., Park W.J., Choi M.K. (2010). Anti-obesity and hypolipidemic effects of Lycium chinense leaf powder in obese rats.. J. Med. Food.

[46] Pinky P.D., Pfitzer J.C., Senfeld J. (2022). Recent insights on glutamatergic dysfunction in alzheimer’s disease and therapeutic implications.. Neuroscientist.

[47] Conway M.E. (2020). Alzheimer’s disease: Targeting the glutamatergic system.. Biogerontology.

[48] Ho Y.S., Yu M.S., Yik S.Y., So K.F., Yuen W.H., Chang R.C.C. (2009). Polysaccharides from wolfberry antagonizes glutamate excitotoxicity in rat cortical neurons.. Cell. Mol. Neurobiol..

[49] Andrade L.J.O., Oliveira L.M., Bittencourt A.M.V., Lourenço L.G.C., Oliveira G.C.M. (2024). Brain insulin resistance and Alzheimer’s disease: A systematic review.. Dement. Neuropsychol..

[50] Cai H., Liu F., Zuo P., Huang G., Song Z., Wang T., Lu H., Guo F., Han C., Sun G. (2015). Practical application of antidiabetic efficacy of *Lycium barbarum* polysaccharide in patients with type 2 diabetes.. Med. Chem..

[51] He Y., Wang Y., Li X., Qi Y., Qu Z., Hu Y. (2024). *Lycium barbarum* polysaccharides improves cognitive functions in icv-stz-induced alzheimer’s disease mice model by improving the synaptic structural plasticity and regulating irs1/pi3k/akt signaling pathway.. Neuromolecular Med..

[52] Domin H., Burnat G. (2024). mGlu4R, mGlu7R, and mGlu8R allosteric modulation for treating acute and chronic neurodegenerative disorders.. Pharmacol. Rep..

[53] Secondo A., Bagetta G., Amantea D. (2018). On the role of store-operated calcium entry in acute and chronic neurodegenerative diseases.. Front. Mol. Neurosci..

[54] Mishra Y., Kumar A., Kaundal R.K. (2025). Mitochondrial dysfunction is a crucial immune checkpoint for neuroinflammation and neurodegeneration: Mtdamps in focus.. Mol. Neurobiol..

[55] Feigin V.L., Brainin M., Norrving B., Martins S., Sacco R.L., Hacke W., Fisher M., Pandian J., Lindsay P. (2022). World stroke organization (WSO): Global stroke fact sheet 2022.. Int. J. Stroke.

[56] Feigin V.L., Owolabi M.O., Feigin V.L., Abd-Allah F., Akinyemi R.O., Bhattacharjee N.V., Brainin M., Cao J., Caso V., Dalton B., Davis A., Dempsey R., Duprey J., Feng W., Ford G.A., Gall S., Gandhi D., Good D.C., Hachinski V., Hacke W., Hankey G.J., Ishida M., Johnson W., Kim J., Lavados P., Lindsay P., Mahal A., Martins S., Murray C., Nguyen T.P., Norrving B., Olaiya M.T., Olalusi O.V., Pandian J., Phan H., Platz T., Ranta A., Rehman S., Roth G., Sebastian I.A., Smith A.E., Suwanwela N.C., Sylaja P.N., Thapa R., Thrift A.G., Uvere E., Vollset S.E., Yavagal D., Yaria J., Owolabi M.O., Owolabi M.O., Feigin V.L., Abd-Allah F., Abera S.F., Akinyemi R., Brainin M., Caso V., Dempsey R.J., Ford G.A., Gall S., Gandhi D., Hachinski V., Hacke W., Hankey G.J., Ibrahim N.M., Johnson W., Lavados P., Liu L., Lindsay P., Martins S., Norrving B., Olaiya M.T., Ovbiagele B., Pandian J., Phan H., Piradov M., Platz T., Ranta A., Roth G., Sebastian I.A., Suwanwela N., Sylaja P.N., Thrift A.G., Uvere E., Yaria J., Abanto C., Addissie A., Adeleye A.O., Adilbekov Y., Adilbekova B., Adoukonou T.A., Aguiar de Sousa D., Akhmetzhanova Z., Akpalu A., El Alaoui-Faris M., Ameriso S.F., Andonova S., Arsovska A., Awoniyi F.E., Bakhiet M., Barboza M.A., Basri H., Bath P.M., Bereczki D., Beretta S., Berkowitz A.L., Bernhardt J., Berzina G., Bhavsar B., Bisharyan M.S., Bohara M., Bovet P., Budincevic H., Cadilhac D.A., Čerimagić D., Charway-Felli A., Chen C., Chin J.H., Christensen H., Chwojnicki K., Conforto A.B., Correia M., Mora Cuervo D.L., Członkowska A., D’Amelio M., Danielyan K.E., Davis S., Demarin V., Demchuk A.M., Dichgans M., Dokova K., Donnan G., Duran J.C., Ekeng G., Elkind M.S., Endres M., Fischer U., Flomin Y., Gankpe F., Gavidia M., Gaye Saavedra A., Gebreyohanns M., George M., Gierlotka M., Giroud M., Gnedovskaya E.V., Gonçalves I.P., Gongora-Rivera F., Gunaratne P.S., Hamadeh R.R., Hamzat T.K., Heldner M.R., Ibrahim E., Ihle-Hansen H., Jee S., Jiann-Shing J., Johnston S.C., Jovanovic D., Jurjāns K., Kalani R., Kalkonde Y., Kamenova S., Karaszewski B., Kelly P., Kiechl S., Kondybayeva A., Kõrv J., Kozera G., Kravchenko M., Krespi Y., Krishnamurthi R., Kruja J., Kutluk K., Langhorne P., Law Z.K., Lebedynets D., Lee T-H., Leung T.W., Liebeskind D.S., López-Jaramillo P., Lotufo P.A., Machline-Carrion M.J., Maia L.F., Malojcic B., Markus H.S., Marquez-Romero J.M., Medina M.T., Medukhanova S., Mehndiratta M.M., Miglāne E., Mihejeva I., Mikulik R., Mirrakhimov E., Mohl S., Munakomi S., Murphy S., Musa K.I., Nasreldein A., Nogueira R.G., Nolte C.H., Noubiap J.J., Novarro-Escudero N., Ocampo C., O’Donnell M., Ogun Y., Ogunniyi A., Oraby M.I., Ōrken D.N., Ōzdemir A.O., Ozturk S., Paccot M., Pereira T., Peeters A., Potpara T., Proios H., Rathore F.A., Sacco R.L., Sahathevan R., Sandset E.S., Renato Santos I., Saposnik G., Sarfo F.S., Sargento-Freitas J., Sharma M., Shaw L., Sheth K.N., Shin Y-I., Shobhana A., Silva S.N., Tedim Cruz V., Thakur K., Thapa L.J., Toni D., Topcuoglu M.A., Torales J., Towfighi A., Truelsen T., Tsiskaridze A., Tulloch-Reid M., Useche J.N., Vanacker P., Vassilopoulou S., Vukorepa G., Vuletic V., Wahab K.W., Wang W., Wijeratne T., Wojtyniak B., Wolfe C., Yacouba M.N., Yang J., Yifru Y.M., Yock-Corrales A., Yonemoto N., Yperzeele L., Zagożdżon P. (2023). Pragmatic solutions to reduce the global burden of stroke: A world stroke organization–lancet neurology commission.. Lancet Neurol..

[57] Seitz R.J., Donnan G.A. (2015). Recovery potential after acute stroke.. Front. Neurol..

[58] Alkahtani R. (2022). Molecular mechanisms underlying some major common risk factors of stroke.. Heliyon.

[59] Yuan Q., Yuan Y., Zheng Y., Sheng R., Liu L., Xie F., Tan J. (2021). Anti-cerebral ischemia reperfusion injury of polysaccharides: A review of the mechanisms.. Biomed. Pharmacother..

[60] Meng H., Jin W., Yu L., Xu S., Wan H., He Y. (2021). Protective effects of polysaccharides on cerebral ischemia: A mini-review of the mechanisms.. Int. J. Biol. Macromol..

[61] Rui C., Yuxiang L., Yinju H., Qingluan Z., Yang W., Qipeng Z., Hao W., Lin M., Juan L., Chengjun Z., Yuanxu J., Yanrong W., Xiuying D., Wannian Z., Tao S., Jianqiang Y. (2012). Protective effects of *Lycium barbarum* polysaccharide on neonatal rat primary cultured hippocampal neurons injured by oxygen-glucose deprivation and reperfusion.. J. Mol. Histol..

[62] Shi Z., Wu D., Yao J.P., Yao X., Huang Z., Li P., Wan J.B., He C., Su H. (2016). Protection against oxygen-glucose deprivation/reperfusion injury in cortical neurons by combining omega-3 polyunsaturated acid with lyciumbarbarum polysaccharide.. Nutrients.

[63] Zhang Q., Jia M., Wang Y., Wang Q., Wu J. (2022). Cell death mechanisms in cerebral ischemia–reperfusion injury.. Neurochem. Res..

[64] Yang D., Li S.Y., Yeung C.M., Chang R.C.C., So K.F., Wong D., Lo A.C.Y. (2012). *Lycium barbarum* extracts protect the brain from blood-brain barrier disruption and cerebral edema in experimental stroke.. PLoS One.

[65] Wang T., Li Y., Wang Y., Zhou R., Ma L., Hao Y., Jin S., Du J., Zhao C., Sun T., Yu J. (2014). *Lycium barbarum* polysaccharide prevents focal cerebral ischemic injury by inhibiting neuronal apoptosis in mice.. PLoS One.

[66] Shi Z., Zhu L., Li T., Tang X., Xiang Y., Han X., Xia L., Zeng L., Nie J., Huang Y., Tsang C.K., Wang Y., Lei Z., Xu Z., So K., Ruan Y. (2017). Neuroprotective mechanisms of *Lycium barbarum* polysaccharides against ischemic insults by regulating NR2B and NR2A containing NMDA receptor signaling pathways.. Front. Cell. Neurosci..

[67] Stanzione R., Pietrangelo D., Cotugno M., Forte M., Rubattu S. (2024). Role of autophagy in ischemic stroke: Insights from animal models and preliminary evidence in the human disease.. Front. Cell Dev. Biol..

[68] Yu Y., Wu X., Pu J., Luo P., Ma W., Wang J., Wei J., Wang Y., Fei Z. (2018). *Lycium barbarum* polysaccharide protects against oxygen glucose deprivation/reoxygenation-induced apoptosis and autophagic cell death *via* the PI3K/Akt/mTOR signaling pathway in primary cultured hippocampal neurons.. Biochem. Biophys. Res. Commun..

[69] Li R., Duan W., Feng T., Gu C., Zhang Q., Long J., Huang S., Chen L. (2023). *Lycium barbarum* polysaccharide inhibits ischemia-induced autophagy by promoting the biogenesis of neural stem cells-derived extracellular vesicles to enhance the delivery of miR-133a-3p.. Chin. Med..

[70] Simats A., Liesz A. (2022). Systemic inflammation after stroke: Implications for post-stroke comorbidities.. EMBO Mol. Med..

[71] Zhao P., Zhou R., Zhu X.Y., Liu G., Zhao Y.P., Ma P.S., Wu W., Niu Y., Sun T., Li Y.X., Yu J.Q., Qian Z.M. (2017). Neuroprotective effects of *Lycium barbarum* polysaccharide on focal cerebral ischemic injury in mice.. Neurochem. Res..

[72] Zhao P., Ma N.T., Chang R.Y., Li Y.X., Hao Y.J., Yang W.L., Zheng J., Niu Y., Sun T., Yu J.Q. (2017). Mechanism of *Lycium barbarum* polysaccharides on primary cultured rat hippocampal neurons.. Cell Tissue Res..

[73] Liu W.J., Jiang H.F., Rehman F.U.L., Zhang J.W., Chang Y., Jing L., Zhang J.Z. (2017). *Lycium barbarum* polysaccharides decrease hyperglycemia-aggravated ischemic brain injury through maintaining mitochondrial fission and fusion balance.. Int. J. Biol. Sci..

[74] Jiang H.F., Guo Y.Q., Rehman F.U., Jing L., Zhang J.Z. (2022). Potential cerebrovascular protective functions of *lycium barbarum* polysaccharide in alleviating hyperglycemia-aggravated cerebral ischemia/reperfusion injury in hyperglycemic rats.. Eur. Rev. Med. Pharmacol. Sci..

[75] Zhao Q., Jing Y.M., He M.T., Jing L., Xi Y.F., Zhang J.Z. (2023). *Lycium Barbarum* polysaccharides ameliorates hyperglycemia-exacerbated cerebral ischemia/reperfusion injury *via* protecting blood-brain barrier.. Transpl. Immunol..

[76] Löscher W., Potschka H., Sisodiya S.M., Vezzani A. (2020). Drug resistance in epilepsy: Clinical impact, potential mechanisms, and new innovative treatment options.. Pharmacol. Rev..

[77] Pong A.W., Xu K.J., Klein P. (2023). Recent advances in pharmacotherapy for epilepsy.. Curr. Opin. Neurol..

[78] Juźwik C.A.S., S Drake S., Zhang Y., Paradis-Isler N., Sylvester A., Amar-Zifkin A., Douglas C., Morquette B., Moore C.S., Fournier A.E. (2019). microRNA dysregulation in neurodegenerative diseases: A systematic review.. Prog. Neurobiol..

[79] Xu X., Mei B., Yang Y., Li J., Weng J., Yang Y., Zhu Q., Zhang H., Liu X. (2025). Astrocytes lingering at a crossroads: Neuroprotection and neurodegeneration in neurocognitive dysfunction.. Int. J. Biol. Sci..

[80] He L.Y., Hu M.B., Li R.L., Zhao R., Fan L.H., He L., Lu F., Ye X., Huang Y., Wu C.J. (2021). Natural medicines for the treatment of epilepsy: Bioactive components, pharmacology and mechanism.. Front. Pharmacol..

[81] Khan A.U., Akram M., Daniyal M., Akhter N., Riaz M., Akhtar N., Shariati M.A., Anjum F., Khan S.G., Parveen A., Ahmad S. (2020). Awareness and current knowledge of epilepsy.. Metab. Brain Dis..

[82] Zhang Y.W., Ma J.B., Sun J.P. (2014). Effect of *Lycium barbarum* polysaccharide influence nerve cell proliferation in hippocampus of epilepsy rat.. Shenjing Jiepouxue Zazhi.

[83] Ma L., Zuo D., Zhang C. (2020). Protective effects of *Lycium barbarum* polysaccharides on epilepsy in rat hippocampal cell model and its mechanism.. Zhongguo Xin Yao Zazhi.

[84] Bloem B.R., Okun M.S., Klein C. (2021). Parkinson’s disease.. Lancet.

[85] Simon D.K., Tanner C.M., Brundin P. (2020). Parkinson disease epidemiology, pathology, genetics, and pathophysiology.. Clin. Geriatr. Med..

[86] Song J., Liu L., Li Z., Mao T., Zhang J., Zhou L., Chen X., Shang Y., Sun T., Luo Y., Jiang Y., Tan D., Tong X., Dai F. (2022). *Lycium barbarum* polysaccharide improves dopamine metabolism and symptoms in an MPTP-induced model of Parkinson’s disease.. BMC Med..

[87] Wang X., Pang L., Zhang Y., Xu J., Ding D., Yang T., Zhao Q., Wu F., Li F., Meng H., Yu D. (2018). *Lycium barbarum* polysaccharide promotes nigrostriatal dopamine function by modulating PTEN/AKT/mTOR pathway in a methyl-4-phenyl-1,2,3,6-tetrahydropyridine (MPTP) murine model of parkinson’s disease.. Neurochem. Res..

[88] Gao K., Liu M., Cao J., Yao M., Lu Y., Li J., Zhu X., Yang Z., Wen A. (2014). Protective effects of *Lycium barbarum* polysaccharide on 6-OHDA-induced apoptosis in PC12 cells through the ROS-NO pathway.. Molecules.

[89] Jia Q., Li S., Li X.J., Yin P. (2022). Neuroinflammation in Huntington’s disease: From animal models to clinical therapeutics.. Front. Immunol..

[90] Pan L., Feigin A. (2021). Huntington’s disease: New frontiers in therapeutics.. Curr. Neurol. Neurosci. Rep..

[91] Gusella J.F., Lee J.M., MacDonald M.E. (2021). Huntington’s disease: Nearly four decades of human molecular genetics.. Hum. Mol. Genet..

[92] Marxreiter F., Stemick J., Kohl Z. (2020). Huntington lowering strategies.. Int. J. Mol. Sci..

[93] Fang F., Peng T., Yang S., Wang W., Zhang Y., Li H. (2016). *Lycium barbarum* polysaccharide attenuates the cytotoxicity of mutant huntingtin and increases the activity of AKT.. Int. J. Dev. Neurosci..

[94] Xing X., Liu F., Xiao J., So K.F. (2016). Neuro-protective mechanisms of *Lycium barbarum*.. Neuromolecular Med..

[95] Li H., Ding F., Xiao L., Shi R., Wang H., Han W., Huang Z. (2017). Food-derived antioxidant polysaccharides and their pharmacological potential in neurodegenerative diseases.. Nutrients.

[96] Fontana L., Ghezzi L., Cross A.H., Piccio L. (2021). Effects of dietary restriction on neuroinflammation in neurodegenerative diseases.. J. Exp. Med..

[97] Liu H., Cui B., Zhang Z. (2022). Mechanism of glycometabolism regulation by bioactive compounds from the fruits of *Lycium barbarum*: A review.. Food Res. Int..

[98] Ding Y., Chen D., Yan Y., Chen G., Ran L., Mi J., Lu L., Zeng X., Cao Y. (2021). Effects of long-term consumption of polysaccharides from the fruit of *Lycium barbarum* on host’s health.. Food Res. Int..

[99] Kwok S.S., Bu Y., Lo A.C.Y., Chan T.C.Y., So K.F., Lai J.S.M., Shih K.C. (2019). A systematic review of potential therapeutic use of *lycium barbarum* polysaccharides in disease.. BioMed Res. Int..

[100] Gong H., Li W., Sun J., Jia L., Guan Q., Guo Y., Wang Y. (2022). A review on plant polysaccharide based on drug delivery system for construction and application, with emphasis on traditional Chinese medicine polysaccharide.. Int. J. Biol. Macromol..

[101] Ye D., Zhao Q., Ding D., Ma B.L. (2023). Preclinical pharmacokinetics-related pharmacological effects of orally administered polysaccharides from traditional Chinese medicines: A review.. Int. J. Biol. Macromol..

